# Investigation of Hf/Ti bilayers for the development of transition-edge sensor microcalorimeters

**DOI:** 10.3762/bjnano.15.108

**Published:** 2024-11-06

**Authors:** Victoria Yu Safonova, Anna V Gordeeva, Anton V Blagodatkin, Dmitry A Pimanov, Anton A Yablokov, Andrey L Pankratov

**Affiliations:** 1 Nizhny Novgorod State Technical University n.a. R.E. Alekseev, Minin Street, 24, Nizhny Novgorod, Russia, 603155, Russia; 2 Institute for Physics of Microstructures of the Russian Academy of Sciences, Akademicheskaya Street, 7, Nizhny Novgorod, Russia, 603950, Russia

**Keywords:** hafnium, microcalorimeter, neutrino, superconducting transition width, superconductivity, TES

## Abstract

The superconducting properties of 85 nm thick hafnium thin films with a 5 nm thick titanium layer on top have been investigated for three different geometries, that is, a film covering the entire 7 × 7 mm^2^ chip surface, bridges with a width of 200 μm and length up to 1800 μm, and bridges in the form of squares with sides from 100 to 1000 μm. The bridges were formed by a photolithographic lift-off process and are intended to be used as the main sensing element of a microcalorimeter based on a transition-edge sensor (TES) in experiments to determine the magnetic moment of neutrinos. Based on the measurements of the critical current, the critical temperature, and the width of the superconducting transition, we estimate the energy resolution δ*E* of the TES prototypes, showing that it is possible to fabricate microcalorimeters with δ*E* less than 1 eV using these films.

## Introduction

Over the last two decades, cryogenic microcalorimeters have found applications in various fields, for example, for the detection of dark matter, as single-photon detectors (X-ray, visible, and infrared ranges) [1], and the detection of individual 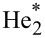 excimers [2]. One of the new applications is the detection of the recoil energy of ^4^He atoms evaporated from a superfluid condensate (helium II). This would allow for the study of the interaction of superfluid helium with neutrinos [3–4]. It is believed that as a result of neutrino scattering from a tritium source on ^4^He in the superfluid state, excitations with energies in the range of 0.1−10 meV arise in the condensate. There is a non-zero probability that these excitations will result in the vaporization of one or more helium atoms from the liquid surface. The vaporized atoms then strike the surface of the microcalorimeter, whose task is to determine their recoil energy. When such atoms are adsorbed on the surface of metals, in addition to the recoil energy, the adsorption energy is released, which for the most common metals reaches tens of millielectronvolts, amplifying the signal. Thus, the task of detecting the neutrino spectrum requires microcalorimeters with an energy resolution capable of distinguishing the initial recoil energy transferred from neutrinos to helium against the background of binding energies, that is, not worse than 0.1 meV. However, a lower resolution is sufficient to detect that a scattering event has taken place, it is estimated that δ*E* of less than 1 eV will already suffice for this purpose.

Microcalorimeters based on a transition-edge sensor (TES) are the most common concept [5]. The developed detectors have an energy resolution of the order of a few electronvolts, which is insufficient for many important applications. At present, the work on increasing the sensitivity of TES-based microcalorimeters is ongoing, and the choice of a superconductor material plays an important role here [6–7].

An increase in sensitivity can be achieved by lowering the critical temperature *T*_C_ below 100 mK. A known solution is two- or multilayer films of various superconductors and normal metals to suppress the transition temperature to the required values because of the proximity effect. One of the most common pairs is titanium with gold [1,8]. Moreover, there are at least two materials that in pure form already possess *T*_C_ close to the required value, iridium with a critical temperature of 112 mK [9] and hafnium with one of about 128 mK [10–11].

The following advantages of Hf can be outlined. It is known that the energy resolution of a microcalorimeter is proportional to the square root of the heat capacity, which depends on the TES volume. Therefore, minimization of the layer thickness is essential for increasing the sensitivity of the microcalorimeter. Our Hf films demonstrate superconductivity at half of the thickness of Ir films [12], allowing for the reduction of the detector volume. Moreover, hafnium is inexpensive in comparison with iridium. Another point is that the technology of Hf deposition, developed in our group, yields predictable and repeatable properties of films, whereas iridium is demanding regarding the deposition conditions: A temperature of the substrate of the order of several hundreds degrees Celsius must be maintained to obtain superconducting films [13–14]. Furthermore, the heat capacitance of Hf is lower than that of Ti, another widely used TES material. This means that the same amount of energy will give a higher temperature change in hafnium than in titanium-based TES.

The listed points make hafnium a promising material for TES development. In this work, Hf/Ti bilayer bridges of different geometries were investigated in a continuation of [10], in which thin films of both pure hafnium and hafnium in combination with normal metals were considered. It is expected that an additional processing operation (lithography and lift-off process) to form the bridges may degrade their properties compared to the full-size films. This is because at the edges of the structure, the film thickness may differ from that at the center. Smooth edges are also necessary for good contact with subsequent layers. For this reason, special attention is paid to the development of technology for creating structures with smooth and even edges during the manufacturing of TES.

The presented measurements are necessary for calculating the sensitivity of future devices and their optimization. In the last section, we evaluate the energy resolution of TES and show that the measured Hf/Ti bilayers, because of the narrow width of the superconducting transition, can be used to fabricate a TES microcalorimeter.

## Sample Fabrication

**Figure 1:**
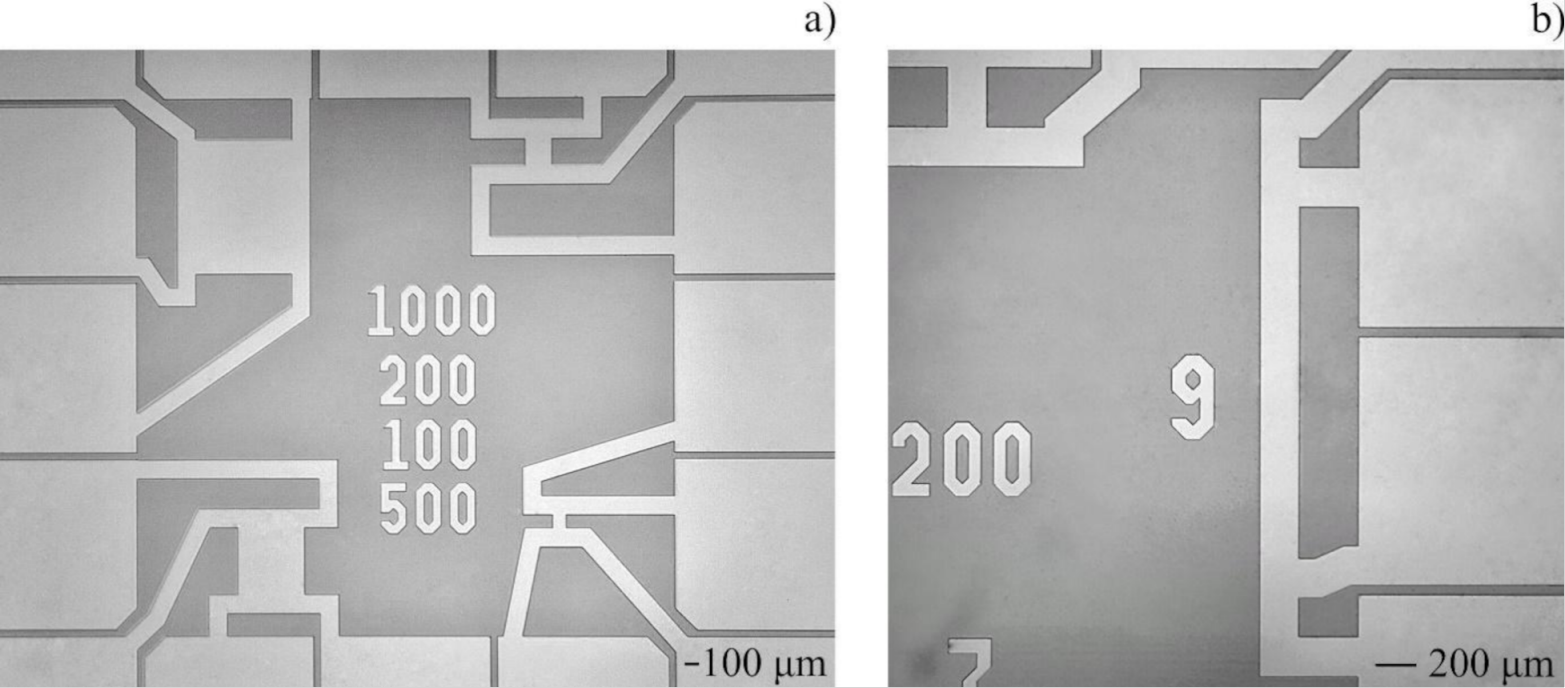
Photographs of structures taken with an optical microscope. (a) Chip with samples A1–A4 and (b) sample B1.

The most typical geometry of a TES is a square, which is advantageous over the elongated shapes for its compactness, leading to more uniform heating during signal readout, and for reduced probability of obtaining inhomogeneous properties along the film during fabrication. As an example, the record energy resolution of just 0.1 eV was demonstrated in TES with sides of 10 × 10 μm^2^ in [15]. We fabricated three types of samples, namely (1) bridges in square shape with sides from 100 to 1000 μm as a TES prototype (A1–A4), (2) films deposited in the form of bridges with different width and length (B1), and (3) films covering the entire substrate (C1). Optical images of the four square bridges and one long bridge investigated in this paper are shown in Figure 1a and Figure 1b.

All investigated samples with bridges of different shapes and lengths were fabricated simultaneously in an electron beam evaporator at a vacuum of 5 × 10^−8^ Torr. The sample with the film was made separately but with the same parameters, including the thickness and evaporation rates.

**Figure 2:**
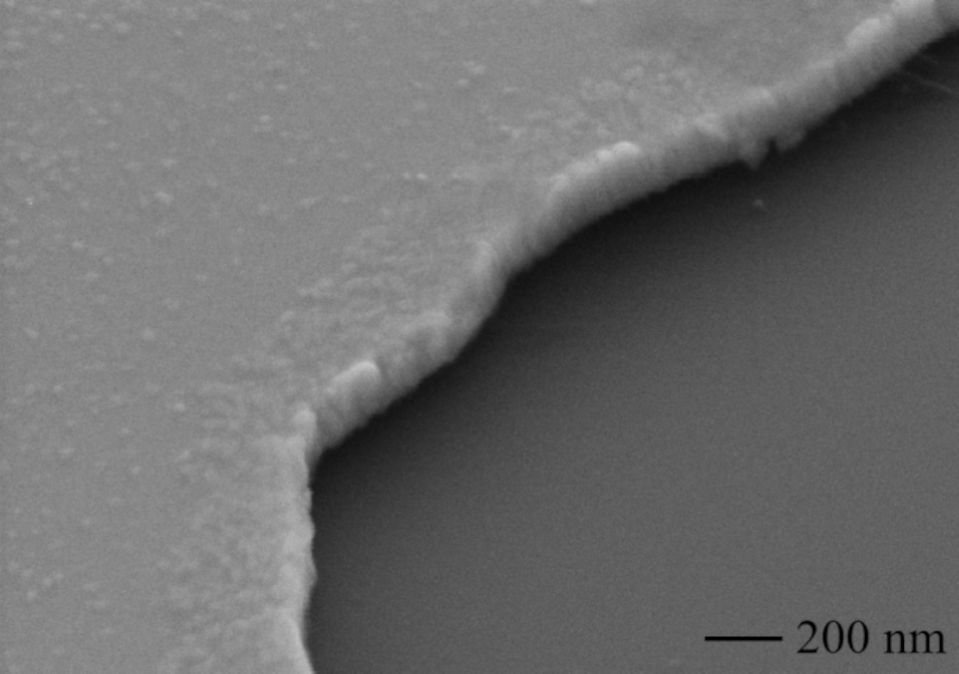
SEM image of the edge of the Hf/Ti bridge structure made with the lift-off process.

On the bridged samples, photolithography was performed on a Karl Suss MJB3 lithography aligner before deposition. We used AZ5214E photoresist, which was subsequently developed with MIF726. We then deposited 85 nm of hafnium and 5 nm of titanium onto the substrates through developed areas in the resist, using an electron beam evaporator. Finally, we performed a lift-off process using *N*-methylpyrrolidone followed by a rinse in isopropyl alcohol. The edges of the structures after the lift-off process appeared to be vertical well-defined walls without upward bends, as shown in the SEM image in Figure 2.

A thin layer of titanium on top of hafnium makes the film properties, such as the critical temperature and the shape of the superconducting transition, stable over time, which is necessary for successful operation of the detectors. For hafnium, the optimal growth rate was 2 Å/s. Films deposited at this rate have a roughness of about 1 nm. Titanium was deposited on hafnium at a growth rate of 0.5 Å/s. The roughness of hafnium films coated with titanium decreased compared to hafnium-only films. Because of the slow deposition rate of Ti, small crystallites with sizes below 10 nm are visible in Figure 2.

The A1–A4 samples of square geometry, shown in Figure 1a, are connected by 140 μm-wide electrodes, which increase the effective bridge length. This has to be thought about when calculating the resistivity. Thus, 280 μm was added to the length of all square bridges except sample A1 (electrodes are already integrated into this sample). The resistivity estimated considering the width of the electrodes ranged from 0.7 × 10^−7^ to 1.1 × 10^−7^ Ω·m for the four structures A1–A4 from Figure 1a. Bearing in mind that the residual resistance ratio of the studied samples is in the range from 1.9 to 2.5, the room temperature resistivity is close to the value of the bulk resistivity at room temperature of 3.3 × 10^−7^ Ω·m [11].

The variation of calculated resistivity between bridges of different sizes is likely not due to physically different film properties, but rather due to rough estimations not taking into account the edge effects for the current flow. Otherwise, the critical temperature of the structures would vary significantly, which we do not observe, as shown below.

## Cryogenic Measurement Results

Low-temperature measurements of the chips with structures were performed in a Triton 200 dilution cryostat. The chips were placed in a 16-pin sample holder with a pin spacing of 1.4 mm. The entire area of the chip was 7 × 7 mm^2^.

**Figure 3:**
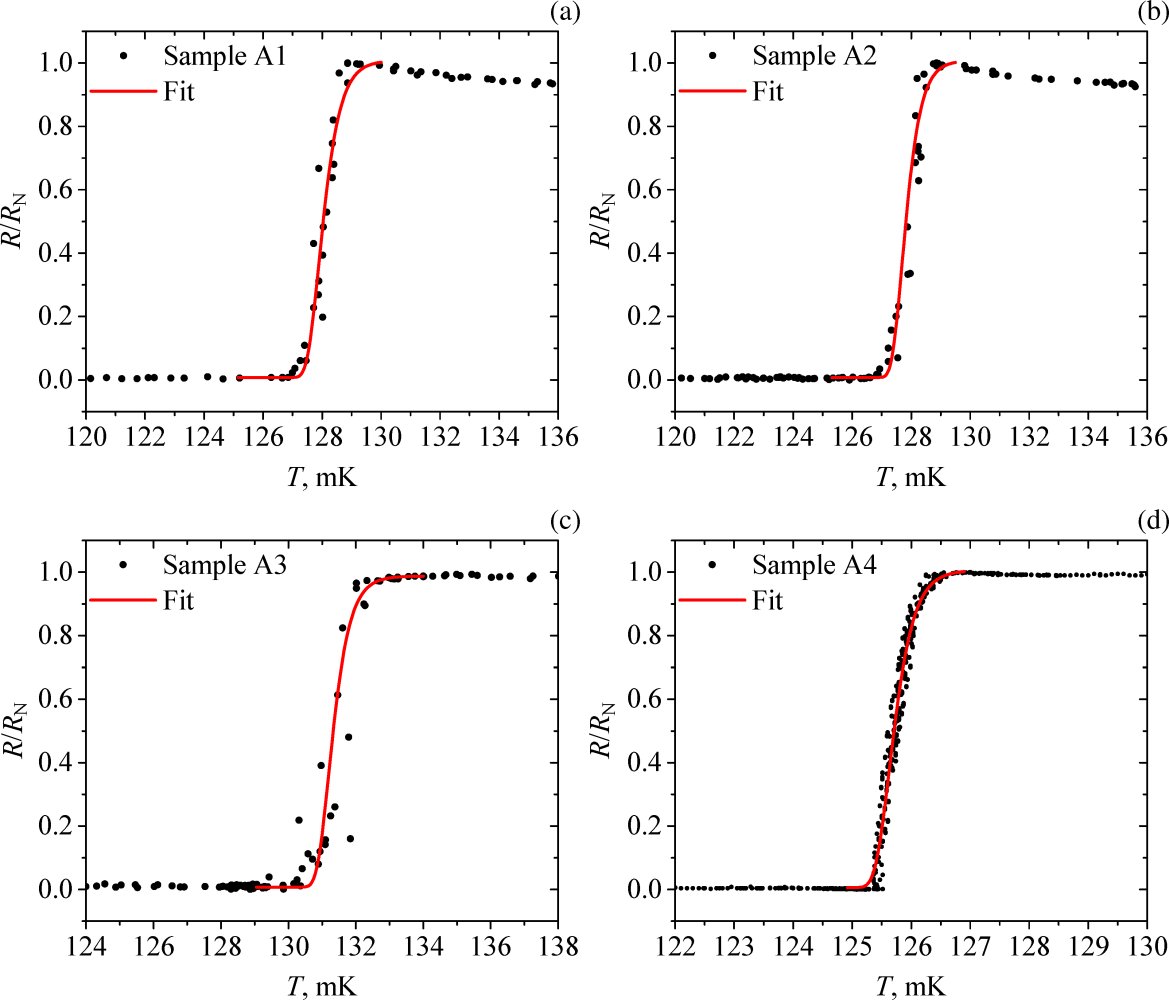
The dependence of resistance versus temperature (black dots) for the test samples: (a) A1, (b) A2, (c) A3, and (d) A4. The red curves show the fitting functions.

The resistance as a function of the temperature *R*(*T*) for samples A1–A4, B1, and C1 has been measured. The parameters of the measured samples are summarized in Table 1. The critical temperature is determined as the temperature where the resistance declines by 50% and the width of the superconducting transition is defined as the temperature interval where the resistance rises from 10% to 90% of the resistance in the normal state. The measured *R*(*T*) dependence of the samples A1–A4 together with the fitting curves is shown in Figure 3.

**Table 1:** Parameters of the studied samples. *I*_C_ is the critical current, *R*_N_ is the resistance in the normal state, *T*_C_ is the critical temperature, δ*T* is the width of the superconducting transition, and *I*_bias_ is the bias current at which we measured the transition.

Sample	Size [μm^2^]	*I*_C_ [μA]	*R*_N_ [Ω] at 30 mK	*R*_N_ [Ω] at 300 K	*T*_C_ [mK]	δ*T* [mK]	*I*_bias_ [μA]

A1	1000 × 1000	1.6	0.6	1.4	128.0	1.2	0.5
A2	500 × 780	6.1	1.6	3.3	128.0	1.1	0.5
A3	200 × 480	2.3	2.5	5.8	131.0	1.0	0.2
A4	100 × 380	6.3	4.3	8.2	125.7	0.5	1.0
B1	1800 × 200	2.7	23	57	128.5	2.0	0.5
C1	7000 × 7000	27	0.2	14	122	2.0	1.0

### Samples A1–A4

One can see from Table 1 and Figure 3 that the width of the superconducting transition in all the measured samples A1–A4 of square geometry decreases with the size of the structure. The smallest sample A4 (Figure 3d) exhibited the narrowest transition. This bridge has been measured with more points and slower temperature change, since it has the sharpest transition. The closest to it in terms of transition width is sample A3 (Figure 3c). Despite the data is less detailed, one still can see that the transition width does not exceed 1 mK.

We also note that the slightly higher critical temperature of sample A3 compared to the others is due to the lower measurement current. The resistance peak on the *R*(*T*) dependence for samples A1 and A2 (Figure 3a and Figure 3b) may appear due to non-equilibrium effects and the presence of NS borders in the setup [16–17]. But presently, we have no detailed picture of this effect.

### Comparison with samples B1 and C1

**Figure 4:**
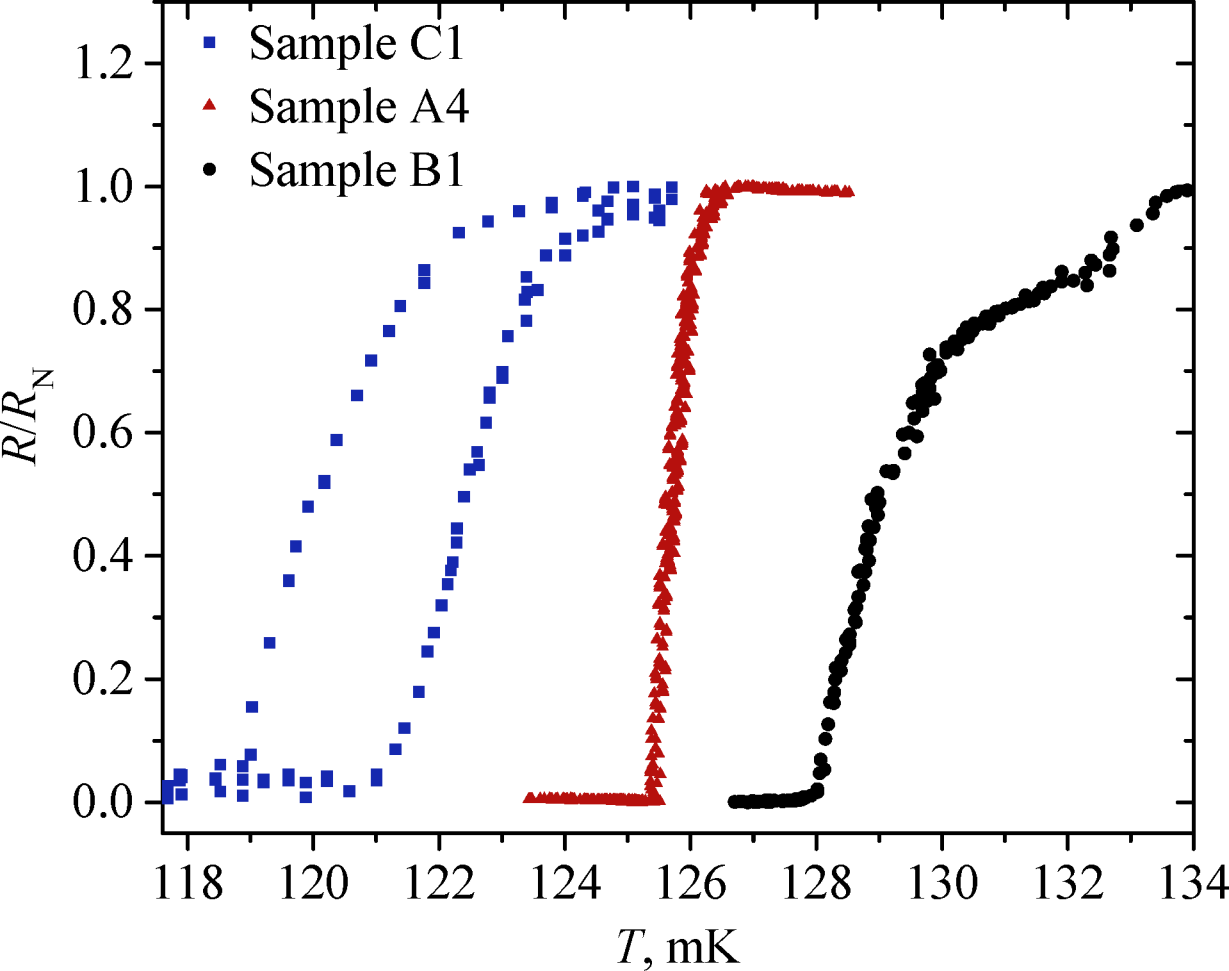
The temperature dependence of resistance of sample C1 (blue squares), sample A4 (red triangles), and sample B1 (black dots). The transition widths of samples B1 and C1 are significantly larger than those of structures A1–A4, which have sharp superconducting transitions without any significant peculiarities.

In this section, we compare the *R*(*T*) dependence obtained for film C1, long bridge B1, and square bridges A1–A4. The resistance as a function of the temperature normalized to the normal resistance is presented in Figure 4 for sample C1 (blue squares), sample A4 (red triangles) and sample B1 (black dots).

All the square bridges A1–A4 performed better than sample B1. The broadening of the transition and the presence of bends in the *R*(*T*) dependence of this bridge are due to inhomogeneities of the film properties, so that in some regions the critical temperature is different. Furthermore, such a protracted transition can be explained by the effect of phase separation in a film of large size, that is, it becomes superconducting in parts.

The film sample C1, unlike samples A1–A4 and B1, shows thermal hysteresis, which is expressed in different transition temperatures at heating and cooling. The presumed reason is the heating of the film sample C1 through the measuring pogo pin contacts made of gold-coated brass and the contact resistance between the contacts and the film. Samples A1–A4 and B1 were measured through the same clamp contacts as sample C1, but the measured area was further away from the contact points. This way, the current first flows through the superconducting electrodes with low thermal conductivity in the superconducting state, and only then goes to the bridge. Nevertheless, non-equilibrium heating effects are still observed in samples A1 and A2, as discussed in the previous section.

## Discussion

**Figure 5:**
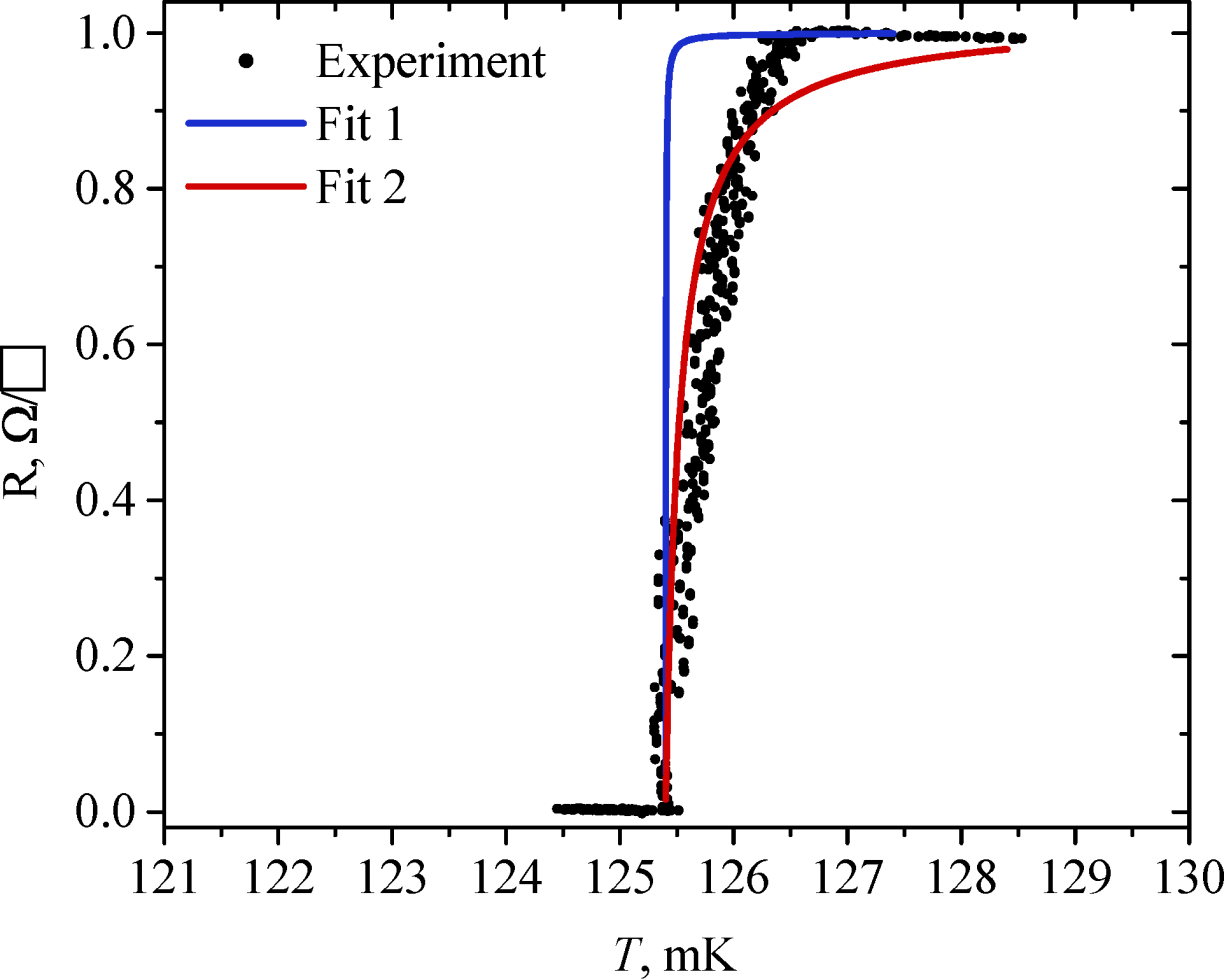
The temperature and resistance relation for sample A4 and its fits with the Aslamazov–Larkin formula.

To answer the question of what determines the transition width of *R*(*T*) of the measured samples, we compare the sharpest transition in structure A4 with the theoretical curve calculated using the Aslamazov–Larkin formula [18]:


[1] $$R\left(T\right)=R_{\text{S}}\left(1-\tau_{0}/\tau_{\text{T}}\right),$$

where τ_0_ is found as (*R*_S_*e*^2^)/(16ℏ), τ_T_ equals to (*T* − *T*_C_)/*T*_C_, and *R*_S_ is the surface resistance of the film. Equation 1 describes the change of film resistance above the critical temperature. This change happens because of thermal fluctuations of conductivity and depends on the surface resistance of the material and its critical temperature only. For sample A4, *R*_S_ is 1 Ω/*□* and τ_0_ is 1.5 × 10^−5^. As the critical temperature *T*_C_, we take the value of 125.4 mK, where *R* becomes zero. The curve with these parameters is shown in Figure 5 (blue curve). As can be seen, it decreases much sharper than the experimental curve. Furthermore, in Figure 5, the red curve is plotted for the τ_0_ value of 1 × 10^−3^; it is close to the experimental points in the resistance range from 0 to 0.9*R*_S_, but deviates from them above 0.9*R*_S_. Thus, the measured dependence *R*(*T*) cannot be properly described by Equation 1 even if we take τ_0_ as a fitting parameter. This means that in addition to the film’s own thermodynamic fluctuations, the transition width and shape are determined by some other factors, for example, current fluctuations, background radiation, and spatial inhomogeneities.

### Temperature sensitivity

According to the measured *R*(*T*) dependence, we have calculated one of the most important parameters for TES, the resistance sensitivity to temperature change α. This parameter determines the ability of a TES to respond to changes in temperature or external signal. In turn, α also depends on temperature and is calculated as the ratio of temperature to resistance multiplied by the derivative of resistance by temperature 
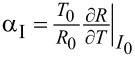
. By analogy, we define the sensitivity of resistance to changes in current 
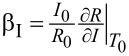
.

For small signals, the TES impedance is represented as a series: 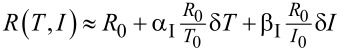, where *R*_0_, *T*_0_, and *I*_0_ stand for the equilibrium values of resistance, temperature, and current, respectively.

**Figure 6:**
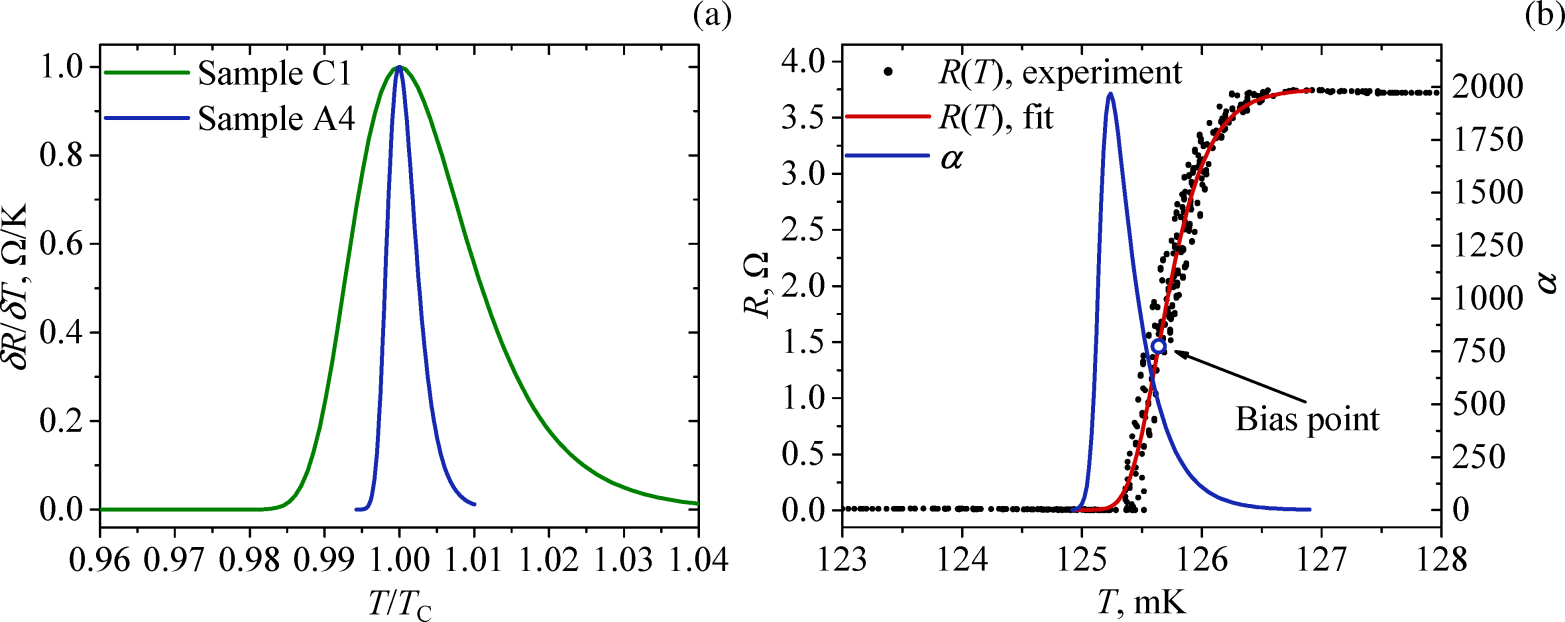
(a) Dependence of normalized derivatives δ*R*/δ*T* versus *T*/*T*_C_ ratio for samples C1 (green curve) and A4 (blue curve). (b) Temperature sensitivity α for sample A4. The black dots are the experimental dependence *R*(*T*). The red curve is the fitting function for *R*(*T*). The blue curve corresponds to α values plotted on the basis of the fitting function. The bias point is the optimal working point for measurements.

Figure 6a shows a comparison of the normalized derivative of resistance by temperature δ*R*/δ*T* versus the ratio of temperature to the critical temperature *T*/*T*_C_ for samples C1 and A4, whose *R*(*T*) dependence is shown in Figure 4. The peak width of this derivative characterizes the sharpness of the transition; the smaller it is, the faster the superconducting transition occurs. As one can see from Figure 6a, the peak width of the derivative for sample A4 is significantly smaller than for sample C1. Consequently, the sharpness of the transition for sample A4 will be higher than for the others.

For sample A4, the calculated temperature sensitivity α is shown in Figure 6b. The highest value of α (almost 2000) is reached at the point of the superconducting transition where the resistance is close to zero, but starts to increase. However, usually, the operating point is chosen in the middle of the transition (for example, the operating bias point is marked with a round marker in Figure 6b, where α takes smaller values of about 500).

In addition to the quality of the deposited film, both the size and the shape of the TES also affect α. Reducing the size of the detector itself increases its sensitivity because of two factors. First, its heat capacity decreases, which is known to determine, along with the operating temperature, the thermodynamic limit of the energy resolution of the microcalorimeter δ*E*^2^ = *k*_B_*T*^2^*C*. A smaller heat capacity generally also means a shorter thermal response time, allowing the detector to respond more quickly to small temperature changes. Second, the active area is reduced, which allows for a more efficient detection of a useful signal because of the higher energy density and, therefore, a higher signal-to-noise ratio.

In addition to the volume, the shape of the TES can also alter the transition width and sensitivity. Comparing sample A1 (Figure 3a) with sample B1 (Figure 4, black dots), one can see that the superconducting transition is much sharper in the square geometry (A1) despite the larger volume of the superconductor. More importantly, the superconducting properties of the structures fabricated by lift-off photolithography not only did not deteriorate compared to continuous films, but became much more suitable for use in TES.

Based on the obtained data on the sensitivity of the detector resistance to temperature change, estimates of the energy resolution of the TES prototype can be made, which are summarized in the next section.

### Estimates of energy resolution

A standard microcalorimeter consists of an absorber, a thermometer, and a thermal coupling to a thermostat. The model from which the microcalorimeter properties are calculated, consists of a thermal body with heat capacity *C* and a thermal coupling to the reservoir with thermal conductance *G*. The thermal body is both an absorber and a thermometer (TES). The quasiparticles of the TES are assumed to be in thermal equilibrium at each instant of time, and their temperature is determined. The applicability of this model is limited by the fact that it does not consider possible inhomogeneities of the film properties and its heating, and the relaxation process is considered as an equilibrium one.

We will use the expressions given in [19] to estimate the TES ampere–watt sensitivity, spectral density of various noise components, and energy resolution. The ampere–watt sensitivity of TES is represented as


[2] $$S_{\text{I}}\left(\omega \right)=-\frac{1}{I_{0}R_{0}}{\left[\begin{array}{l} \frac{L}{\tau_{\text{el}}R_{0}L_{\text{I}}}+\left(1-\frac{R_{\text{L}}}{R_{0}}\right)-\frac{\omega^{2}\tau L}{R_{0}L_{\text{I}}}\\ +i\omega \frac{L\tau }{R_{0}L_{\text{I}}}\left(\frac{1}{\tau_{\text{I}}}+\frac{1}{\tau_{\text{el}}}\right) \end{array}\right]}^{-1}.$$

Here, *L*_I_ is the DC gain, τ is the time constant in the absence of electrothermal feedback, τ_I_ is the time constant in the limit when the current through the TES is constant, τ_el_ is the electrical time constant of the power supply circuit, *L* is the inductance of the input coil, and *R*_L_ is the shunt resistance and parasitic resistance.

The energy resolution δ*E* is the minimum energy difference that can be seen in the microcalorimeter response [20]:


[3] $$\delta E={\left(\int_{0}^{\infty }\frac{4}{{\text{NEP}}^{2}\left(f\right)}\text{d}f\right)}^{-\frac{1}{2}},$$

where NEP is the noise equivalent power. The NEP is equal to the ratio of the total noise power spectral density *I*_N_ to the ampere–watt sensitivity *S*_I_(ω). The total noise is composed of four components, namely, phonon noise 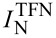, Nyquist TES noise 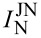, Nyquist external circuit noise 
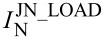
, and the readout electronics (SQUID) noise 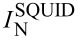. We do not give here expressions for the individual noise components as they are well known and can be found in TES-dedicated papers, for example, in [5,19,21].

We calculate the energy resolution of the TES based on the following parameters of sample A4: the resistance at the operating point *R*_0_ = 1.46 Ω, the plate temperature *T*_0_ = 65 mK, the bias current *I*_0_ = 1 μA, the inductance *L* = 400 nH, the shunt resistance *R*_L_ = 0.3 Ω, the temperature sensitivity α = 500, the current sensitivity β_I_ = (*R*_0_ − *R*_L_)/(*R*_0_ + *R*_L_), the heat capacity *C* = 6 × 10^−14^ J/K, the thermal conductance *G* = 5 × 10^−10^ W/K, calculated for the electron–phonon constant of 0.8 nW/K^6^/μm^3^, and the sixth degree of temperature according to [22].

**Figure 7:**
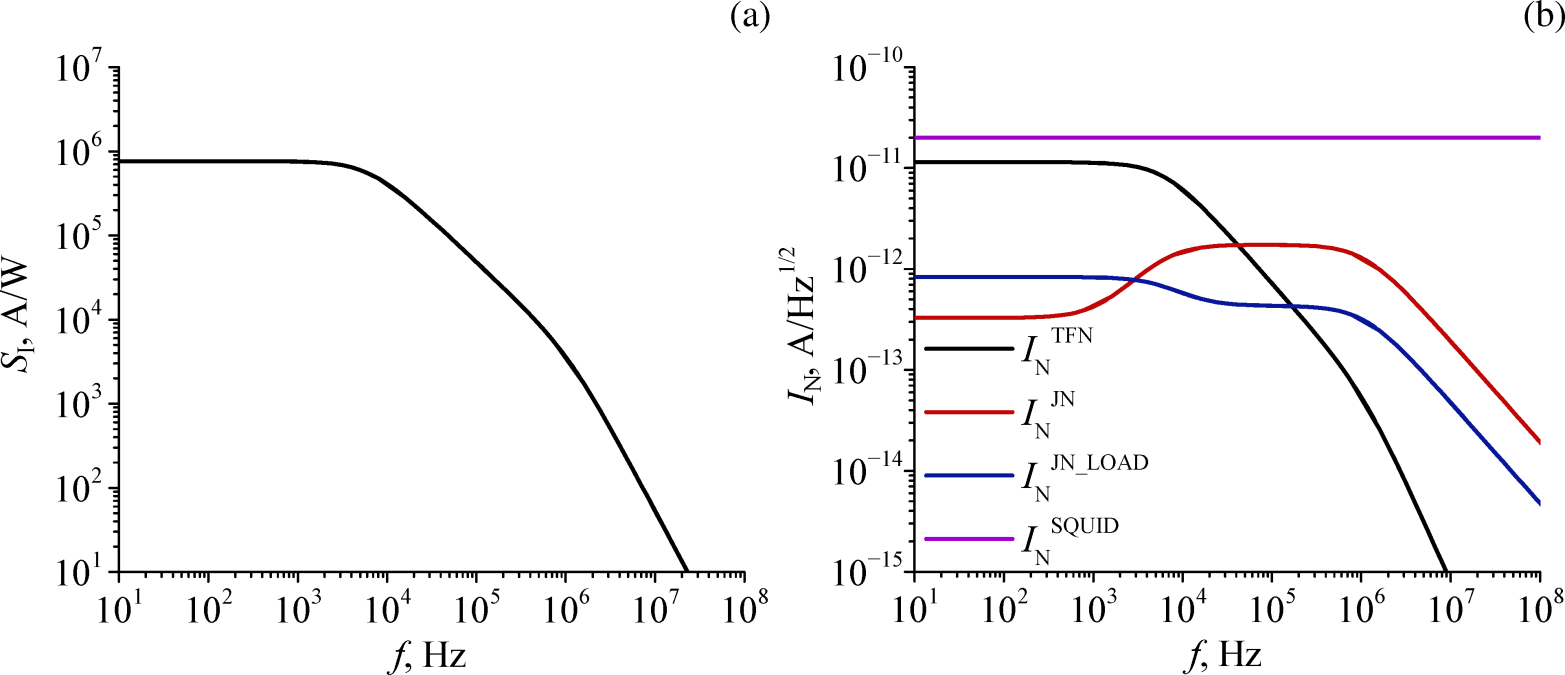
TES characteristics calculated from the parameters of sample A4. (a) Ampere–watt sensitivity and (b) spectral current density of noise components. The calculated energy resolution is about 0.01 eV.

Figure 7 shows the ampere–watt sensitivity and noise characteristics of the TES with above parameters for sample A4. The maximum current response is observed at frequencies from 0 to 1 kHz and is 7.6 × 10^5^ A/W. The current noise of the SQUID readout system from Supracon, available in our lab, is calculated to be 20 pA/Hz^1^*^/^*^2^. It can be seen that the intrinsic noise of the TES and feedback circuit is significantly lower than the noise of the SQUID, so that the total noise is determined mainly by the noise of the SQUID. The energy resolution δ*E* of the TES is expected to be 0.01 eV according to the calculations based on Equation 2 and Equation 3. The ultimate energy resolution of the TES, provided that a low-noise SQUID is used (current noise of the order 1 pA/Hz^1^*^/^*^2^), can reach 0.005 eV.

**Figure 8:**
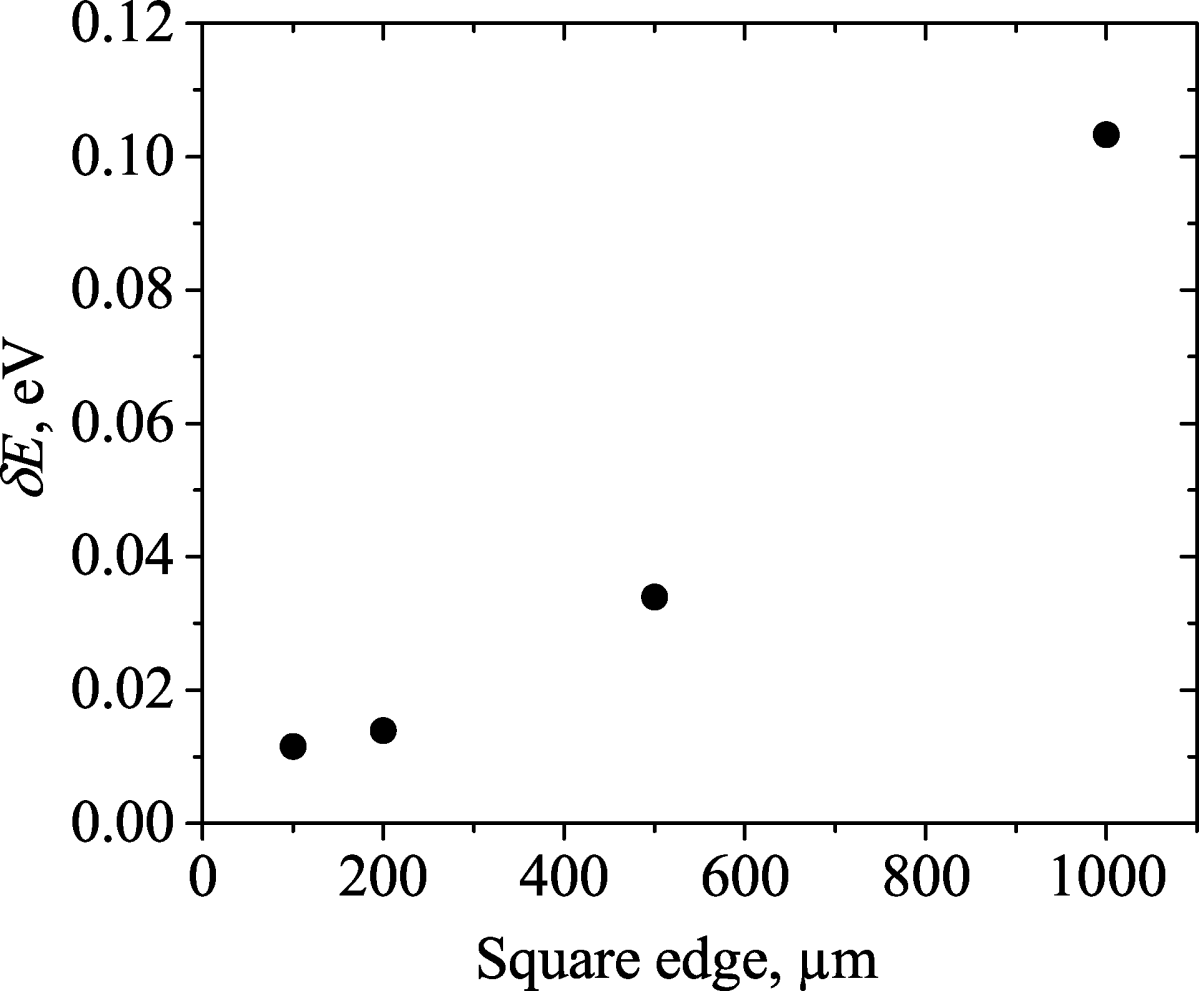
Energy resolution δ*E* as a function of TES square edge length.

Now, we estimate how the energy resolution will deteriorate with increasing TES volume, but with preservation of the square shape. For this purpose, in addition to the already calculated dimensions of sample A4, let us consider several dimensions corresponding to samples A1–A3. For simplicity, we take the parameter α equal to 500 for every calculation and change only the volume-dependent parameters, such as heat capacity and thermal conductance. The dependence of the energy resolution on the edge length of the square TES is shown in Figure 8.

Expectedly, as the detector size increases, its energy resolution deteriorates. Nevertheless, for all calculated sizes, δ*E* turns out to be less than 0.1 eV; only for the sample size A1, it slightly exceeds 0.1 eV. It should be noted that this calculation does not take into account the deterioration of parameter α with increasing TES size, which would lead to an even larger increase of δ*E* with volume.

## Conclusion

Bridges made of bilayers of hafnium with a thin top layer of titanium were fabricated and measured. The formation of such bridges by a photolithographic lift-off process is the next step in the development of TES microcalorimeters after the study of films of these materials carried out in [10]. It is shown that measurements of bridges allow for a more accurate characterization of the material than the measurements of substrate-sized films. Compared to films, bridges exhibit a much sharper transition from the normal to the superconducting state, that is, less than 1.2 mK for bridges compared to more than 2.2 mK for films. The measurements also demonstrate that the quality of the performed lift-off photolithography is sufficient to obtain high values of the temperature coefficient α; hence, this process can be used to fabricate the first TES layer.

The dependence of the superconducting transition sharpness on the bridge size has also been investigated. It indicates that in smaller bridges, the transition occurs more uniformly over the entire area of the structure, while in large bridges and films, because of their larger area, there is an inhomogeneity of the superconducting properties. It is not related to the non-uniformity of cooling of the sample, as it remains regardless of the cooling rate, and leads to an increase in the transition width. Thus, bridges allow us to study the properties of superconducting materials without distorting them by macroscopic effects as overheating and inhomogeneities.

The investigated microbridges will be used to fabricate TES-based microcalorimeters. The energy resolution is estimated to be much better than 1 eV. Such detectors are highly demanded in tasks of detecting recoil energy from single quantum vaporized helium atoms, as well as for dark matter search.

## Data Availability

The data that supports the findings of this study is available from the corresponding author upon reasonable request.
